# Inter-Firm Executive Mobility and Corporate Social Responsibility: Evidence From China

**DOI:** 10.3389/fpsyg.2022.904450

**Published:** 2022-06-03

**Authors:** Jun Wang, Jieling Cao

**Affiliations:** School of Business Administration, Northeastern University, Shenyang, China

**Keywords:** corporate social responsibility, executive turnover, inter-firm mobility, behavioral consistency theory, employment gap

## Abstract

The executives of listed firms play an important role in the fulfillment of corporate social responsibility (CSR). Based on behavioral consistency theory, this study examines the association of CSR performance among multiple firms for the same executive served at different times. By tracking the movement of executives across Chinese listed firms over the period 2010–2019, we find that there is a significantly positive association between the predecessor and the successor firm’s CSR performance for the same executive, implying that an individual’s value and preference for CSR maintain consistency within a certain period of time. We also find that a longer employment gap and lower internal control effectiveness will damage the association of CSR performance between the predecessor and the successor firm. Our results are robust to testing in subsamples and controlling the endogeneity problems. Our conclusion provides a new perspective to understand the influence mechanism of CSR performance in the context of inter-firm executive mobility and provides empirical evidence for listed firms to improve their decision-making in hiring and evaluating executives.

## Introduction

Corporate social responsibility (CSR) has become the focus of public attention from practice and academia since the 1980s (Lee and Carroll, 2011). In accordance with McWilliams and Siegel (2001), we define CSR as “actions that appear to further some social good, beyond the interests of firms and that which is required by law”. A remarkable CSR fulfillment can promote a firm’s long-term sustainable development because it is conducive to satisfying the demand of numerous stakeholders including shareholders, employees, customers, suppliers, and local community organizations (Freeman, 1984). To strengthen Chinese listed firms’ attitude to fulfill their social responsibility, China Securities Regulatory Commission (CSRC) introduced guidelines on the social responsibility of listed firms in 2006. However, the overall CSR performance of Chinese listed firms has not met expectations. As a result, it is of great theoretical and practical significance to explore how to enhance the listed firms’ willingness to fulfill CSR and improve the quality of CSR practices in an emerging market (Yin and Zhang, 2012; Rauf et al., 2021).

Executive turnover is necessary for the process of firm development and strategy realization. The statistics in this study show that an average of 32.3% of Chinese listed firms experience executive turnovers each year between 2010 and 2019.^1^ As a major strategic adjustment of the firm, the dismission of old executives and the succession of new ones will have a certain degree of impact on CSR performance (Meng et al., 2013; Bernard et al., 2018; Rauf et al., 2021). However, the above studies only focus on the changes in CSR performance of a single firm before and after executive turnover. For example, Meng et al. (2013) and Bernard et al. (2018) show that the impact of executive turnover on CSR performance differs with the reasons for leaving and the types of succession. Rauf et al. (2021) further provide evidence on the role of corporate political embeddedness in the association between a firm’s executive turnover and the quality of its CSR disclosure. Since executive turnover may lead to inter-firm executive mobility, we can identify the predecessor firm and the successor firm for a certain executive and further explore the resemblance in CSR performance of the same executive’s predecessor and successor firm, which will deepen our understanding of the relationship between executive turnover and CSR performance.

According to behavioral consistency theory in social psychology, individual behavior and decision preference may display certain similarity and consistency in diverse settings (Allport, 1966; Epstein, 1979). In line with this theory, the executives’ idiosyncrasies are influenced by their early experiences to some extent and cause them to make the same or similar behavioral decisions in different situations. A range of empirical findings indicate that cross-firm executives exhibit distinctive styles in corporate policies and operational decisions (Bertrand and Schoar, 2003; Bamber et al., 2010; Dyreng et al., 2010; Ge et al., 2011; Wells, 2019). For these reasons, we believe that the CSR fulfillment is likely to reflect executives’ value and preference for social responsibility which may not vary significantly in the short term. Hence, as executives switch jobs to another firm, their CSR styles will impose an influence on CSR fulfillment and performance of their successor firms.

Nevertheless, adaptation-level theory states that individuals adapt their behavior in response to the changing environmental conditions (Helson, 1964; Wohlwill, 1974). When executives change to work at a new firm, in order to alleviate the stimulation and pressure rising from the new organizational environment, they may adjust their behavior accordingly, thereby reducing threats and enhancing their chances of survival. They may also learn from past failures and proactively change their behavior to improve decision accuracy (Zollo and Singh, 2004; Madsen and Desai, 2010; Gong et al., 2019). Consequently, it is an empirical question whether executives’ CSR fulfillment will remain relatively stable under the influence of their early experiences and personality traits, or will change in compliance with the new organizational environment.

By tracking the movement of executives across Chinese listed firms from 2010 to 2019, we find a significantly positive association between the predecessor and the successor firm’s CSR performance when they are served by the same executive at different times even if they are totally distinct firms, supporting behavioral consistency theory. Furthermore, we find that the association of CSR performance between the predecessor and the successor firm is negatively moderated by a longer employment gap and lower internal control effectiveness. Our results are robust to a series of robustness tests, including subsample tests, Propensity Score Matching (PSM) method, Heckman two-stage model, and Two-Stage Least Squares (2SLS) approach.

Our study contributes to the extant literature in several ways. Firstly, existing researches mainly focus on the determinants and economic consequences of executive turnover from the perspective of a single firm, such as the impact of incoming executives on a firm’s accounting policy choices and financial performance (Moore, 1973; Murphy and Zimmerman, 1993; Pourciau, 1993; Kato and Long, 2006; Chang and Wong, 2009). Only a few studies further explore where executives are re-employed after their departures from the prior employers, however, most of them focus only on executives’ ship jumping behavior from distressed firms (Fee and Hadlock, 2003; Marcel and Cowen, 2014; Jiang et al., 2017). This study extends to investigate the relevance of executives’ decision-making between the predecessor and the successor firm by tracking their movements across Chinese listed firms and finds a strong behavioral consistency between incoming executives’ decisions (especially those related to CSR) in the new firms and their previous work experience, expanding the research perspective of executive turnover studies.

Secondly, a number of studies underline that certain characteristics of a firm play an important role in CSR performance (Roberts, 1992; Artiach et al., 2010; Khan et al., 2013). Some studies also find that executives’ heterogeneity such as demographic characteristics and personality traits will influence their participation in CSR activities (Manner, 2010; Chin et al., 2013; Tang et al., 2015; Petrenko et al., 2016; McGuinness et al., 2017; Davidson et al., 2018; Yuan et al., 2019; Shaheen et al., 2021). However, few studies have examined the impact of executives’ past experience in CSR practice in prior firms on the current firm’s CSR performance. Task-specific human capital theory holds that not all experiences and skills are useful for a person’s job, only relevant ones matter (Gibbons and Waldman, 2004). Custódio et al. (2013) also stress the importance of relevant work experience. In this study, we explore the impact of executives’ specific experience in CSR practice when they held similar positions in the predecessor firms on the CSR performance of the successor firms, enriching the literature on CSR determinants at the individual level.

Our findings also have significant implications for the design of appropriate employment and talent evaluation system for listed firms. Bernard et al. (2018) state that shareholders’ expectations on CEOs are not solely economic and financial but also concern the CSR performance, and they tend to hire new CEOs and urge them to strengthen CSR fulfillment. They also suggest that CEOs should be evaluated on the basis of CSR performance and not just on accounting and stock performance. Our study provides a new perspective to recognize the value of executives’ past experience in CSR practice, which echoes the view of Bernard et al. (2018) and provides empirical evidence for the executive hiring decisions of listed firms.

The remainder of this manuscript is organized as follows. First, the literature review and hypotheses development are discussed. Then, we present the research design, followed by the empirical results, additional analyses, and robustness tests. Finally, we conclude and discuss future research opportunities.

## Literature Review and Hypotheses Development

Corporate social responsibility is generally considered to be a firm’s voluntary activities on social, environmental, and ethical issues (Carroll, 1999). Over the past two decades, many studies focus on the drivers of a firm’s CSR performance. At the firm level, high profitability and better financial performance allow a firm for superior CSR performance since they are more affordable (McGuire et al., 1988; Pava and Krausz, 1996). Larger size and higher visibility motivate firms to engage in more CSR activities because they attract greater attention from the public (Chiu and Sharfman, 2011; Dhaliwal et al., 2011; Wang and Qian, 2011). Campbell (2007) interprets the influencing factors of CSR performance at the institutional level and suggests that a firm’s engagement in CSR activities is likely to be affected by the regulations, economic conditions, and industry practices.

As the decision-maker of listed firms, executives are closely related to the fulfillment and performance of CSR. A growing number of literature have focused on how the demographic characteristics and personality traits of corporate executives influence CSR fulfillment. For instance, Chin et al. (2013) examines executives’ political ideologies and finds that compared with conservative CEOs, liberal CEOs exhibit greater advances in CSR performance and tend to emphasize CSR practices even if recent financial situation is relatively poor. Tang et al. (2015) affirm that with overestimation of their own capability, hubristic CEOs are inclined to neglect resource dependence on stakeholders, resulting in a lower degree of engagement in CSR practices. Using a sample of Chinese listed firms, McGuinness et al. (2017) find that the appointments of female officers as senior managers are more likely to realize better CSR outcomes. Researchers also suggest that executives’ narcissism, materialism, and managerial ability significantly influence CSR performance (Petrenko et al., 2016; Davidson et al., 2018; Yuan et al., 2019).

Behavioral consistency theory posits the persistence of individual behavior in different situations (Allport, 1966; Epstein, 1979). By tracking senior managers across different firms over time, Bertrand and Schoar (2003) assert that each executive possesses a unique and stable managerial style, namely manager fixed effects, which usually matter in operational and financial policies. Many researchers follow this study and examine the importance of managerial styles in corporate decisions such as investment and financing decisions, financial information disclosures, and accounting policy choices (Bamber et al., 2010; Dyreng et al., 2010; Ge et al., 2011; Francis et al., 2013; Wells, 2019).

Based on this theory, in addition to the personal characteristics mentioned above, the cognitive and behavioral patterns will also be driven by executives’ professional experience, then influencing the decision-making in other firms (Elsaid et al., 2011; Dittmar and Duchin, 2016; Chen et al., 2017; Georgakakis and Ruigrok, 2017; Enkhtaivan and Davaadorj, 2021). For example, Elsaid et al. (2011) examine that stock market reacts positively to the hiring of an outsider with prior CEO experience. Georgakakis and Ruigrok (2017) argue that outsider CEOs with experience from a variety of industries will be better to transfer diverse industry-specific knowledge to the organization, resulting in superior financial outcomes. Enkhtaivan and Davaadorj (2021) find that the corporate liquidity policy of an executive’s predecessor firm is significantly positively correlated with that of his successor firm. All these studies provide evidence that individual behavior of executives are consistent across different situations.

Given the above discussion, personal value and behavioral preference of executives cultivated or displayed in their previous organizations is likely to persist even if they switch jobs. It will pose a potential influence on their decision-making in the new organizational environment, which causes a certain resemblance between the predecessor and the successor firm in some ways. Therefore, we argue that when an executive moves to another firm, his value and preference for CSR reflected in the predecessor firm will maintain consistency and lead to similar decisions and practices about CSR at the successor firm. Given these arguments, we put forward a hypothesis as follows:

**Hypothesis 1:** Ceteris paribus, there is a significant positive association between the CSR performance of the predecessor and the successor firm for the same executive.

There is usually an interval of time during an executive’s position change between different firms, namely, the employment gap. Understanding executive’s employment gap is important because the prevalence and the length of employment gaps indicate a certain amount of frictions in the labor market (Ertimur et al., 2018). In China, the phenomenon of executive’s employment gap is very common. The statistical results in this study show that 60.1% of executives experience an employment gap in the process of inter-firm mobility, with an average duration of 1.4 years from 2010 to 2019.

Marquis and Tilcsik (2013) suggest that the persistence of an individual’s behavior gradually decays over time. Since the employment gap is often accompanied by work interruptions, it is more likely to lead to a deterioration in the consistency of individual behavior. Previous studies indicate that the presence and the length of the employment gap has a significant impact on the quality of executive-firm matching as well as executive compensation in the successor firms (Edin and Gustavsson, 2008; Kroft et al., 2013; Ertimur et al., 2018). Chen et al. (2017) find that the persistence of earnings management behavior of executives also decreases as the employment gap between their tenures at the two firms increases.

In addition, reinforcement learning theory proposes that repetition can promote the formation of individual reflexive behavior, enhance the mastery of knowledge, and improve confidence (Erev and Roth, 1998). Hence, executives’ decision-making can also be reinforced by continuous repetition. For example, Dittmar and Duchin (2016) find that CEOs who experience continual financial distress in their predecessor firms are likely to make more conservative financial policies in their successor firms. Edin and Gustavsson (2008) also show that sitting out of the job market can lead to a decline in executives’ skills as they have not been used and updated for some time. As a result, for executives who experience a longer employment gap and have not made similar decisions for a long time, their decisions would be less influenced by the professional experience in the predecessor firms when they subsequently encounter similar situations at the successor firms.

In conclusion, we argue that in the context of executives’ inter-firm mobility, a longer employment gap would inhibit the executives’ behavioral consistency on the CSR fulfillment, which in turn weakens the positive association between the CSR performance of the predecessor and the successor firm. Given these arguments, we put forward a hypothesis as follows:

**Hypothesis 2:** Ceteris paribus, a longer employment gap weakens the positive association between the CSR performance of the predecessor and the successor firm for the same executive.

As stated in adaptation-level theory, individual behavior will change according to the changing environment (Helson, 1964; Wohlwill, 1974). As a result, in addition to the moderating effect of the employment gap which is a feature at the individual level, the differences in some firm-level characteristics of the predecessor and the successor firm may also affect the relationship between the CSR performance of these two firms.

Since fulfilling CSR means that firms undertake multiple social responsibilities to numerous stakeholders, they will try to create a public image of compliance with laws and regulations, transparency, and profit maximization while sustainable development. In order to achieve this goal, the firms need to establish a corresponding internal system (Hao et al., 2018; Li et al., 2018). As an important and comprehensive institutional arrangement of listed firms, the internal control system can effectively avoid business risks by supervising and correcting the production and operation process (Spira and Page, 2003; Doyle et al., 2007).

In recent years, Chinese listed firms are attaching importance to the construction of internal control systems to achieve sustainable development. In the “*Application Guidelines No. 4 of Enterprise Internal Control—Social Responsibility*” issued by the Ministry of Finance of China, it is specified that listed firms should fulfill their social responsibility and obligations, mainly including safety production, product quality, environmental protection, and employment promotion, which implies that CSR should be considered as a part of internal control. Therefore, it is an important function of internal control to supervise the fulfillment of CSR and safeguard the legitimate rights and interests of stakeholders.

Previous studies also provide empirical evidence that an effective internal control system can improve CSR performance because it prevents misconduct that damage corporate reputation and public image, thereby controlling social responsibility risks and promoting the successful realization of the strategic goals of CSR practice (Hao et al., 2018; Li et al., 2018). Kim et al. (2017) find that CSR firms are more likely to have an effective internal control system and less likely to have material internal control weaknesses. Moreover, internal control is generally considered as an integral component of corporate governance (Hoitash et al., 2009). Compared with firms with inferior corporate governance which are more susceptible to a material internal control weakness, a well-governed firm performs better in social responsibility due to the advantages in CSR initiatives, information processing, management monitoring, and other corporate behaviors (Johnson and Greening, 1999; Jo and Harjoto, 2011; Lau et al., 2016).

In conclusion, we argue that in the context of executives’ inter-firm mobility, the successor firm’s more effective internal control system than the predecessor firm could reduce executives’ risk-taking behaviors and raise their initiatives to engage in CSR activities in the new working environment, which in turn strengthens the positive association between the CSR performance of the predecessor and the successor firm. Given these arguments, we put forward a hypothesis as follows:

**Hypothesis 3:** Ceteris paribus, a higher level of internal control system strengthens the positive association between the CSR performance of the predecessor and the successor firm for the same executive.

## Research Design

### Data Source and Sample Selection

The sample examined in this manuscript includes Chinese listed firms publicly traded in Shanghai and Shenzhen Stock Exchanges and all their executives (including directors, supervisors, and senior management) disclosed in the annual reports. The sample period is 2010–2019 due to the data availability in the database. The data on listed firms’ executive information and other financial and fundamental characteristics used in this manuscript are from the China Stock Market Accounting Research (CSMAR) database. The database contains a list of all executives for each listed firm each year, from which we can observe an executive’s tenures in all listed firms and further identify an executive’s inter-firm mobility (if any). The CSR performance data and internal control index are obtained from Hexun.com and Shenzhen Dibo Internal Control and Risk Management (DIB) database.

Table 1 reports the sample selection process. Firstly, we construct our data to the “executive-firm-appointment year-departure year” level. We define the first (last) year in which an executive appeared in a listed firm during our sample period as the appointment (departure) year. If an executive was re-employed by a firm after leaving it for a period of time, it constitutes two observations in our sample.^2^ For observations that the executive has been in a firm until the end of the sample period, we further check whether this executive still works at the same firm in the next year. The departure year is null if the executive is still with the firm in the following year. Since we focus on the inter-firm executive mobility, the research sample is limited to executives who have worked in at least two listed firms, with a total of 56,280 observations.

**TABLE 1:** Sample selection process.

	Observations
Sample of executive turnover from 2010 to 2019 with “executive-firm-appointment year-departure year” level data	56,280
Dropped: observations with non-sequential turnover	19,139
Dropped: observations with tenure less than 1 year	2,085
Remaining sample	35,056
Reorganized into “executive-predecessor firm-successor firm” level data	17,528
Number of listed firms involved	3,452
Number of individual executives involved	6,108
Dropped: observations with missing data	1,048
Dropped: singleton observations	537
Final sample	15,943

Secondly, in order to eliminate the influence of concurrent positions of executives in different firms on the results, we drop 19,139 observations with overlapping periods for an executive in the predecessor and the successor firm. In other words, we need to ensure that all executives in our sample worked for the successor firms after they left the predecessor firms, namely, a sequential turnover.^3^ We also drop 2,085 observations that the newly-appointed executive served in the successor firm for less than 1 year because the former executive might have a certain impact on corporate decisions and it might be difficult for the newly-appointed executive to have an impact on the CSR performance in a short tenure.

Thirdly, we reshape the data structure of the remaining 35,056 observations into the “executive-predecessor firm-successor firm” level for the following analyses, resulting in 17,528 (35,056/2) observations. It involves 3,452 listed firms and 6,108 individual executives (including 2,872 executives who move between two firms and 3,236 executives who move between more than three firms during our sample period). Last, we drop another 1,048 observations due to missing values of variables and 537 singleton observations after including fixed effects. The final sample consists of 15,943 observations.

### Variable Definition and Model Construction

#### Dependent and Independent Variables

We define the CSR performance of the successor firm (*P_CSR*) as the dependent variable and the CSR performance of the predecessor firm (*F_CSR*) as the independent variable. In accordance with existing studies (Yang et al., 2019; Lu et al., 2020), we utilize the CSR score and rating from HeXun’s CSR Assessment System for Listed Firms to measure the overall CSR performance. Distinct from the other measures merely relying on the social responsibility report, HeXun’s CSR Assessment System refers to both the social responsibility report and the annual report of listed firms, which ensures that the data is more objective and comparable (Zhong et al., 2019). Specifically, *P_CSR*_*S* equals the CSR score of the successor firm in the first full year of the executive’s employment (/100); *P_CSR*_*R* equals the CSR rating of the successor firm in the first full year of the executive’s employment. The CSR rating is ranging from A (highest) to E (lowest). We define the rating of A as 5, B as 4, and so on. Similarly, the CSR performance of the predecessor firm is measured by the CSR score and rating in the last year of the executive’s employment (*F_CSR*_*S/F_CSR*_*R*). The higher the CSR score and rating, the better the overall CSR performance.

#### Moderator Variables

In this study, we take the employment gap (*GAP*) and internal control quality (*ICQ*) as the moderator variables. Referring to the prior literature (Ertimur et al., 2018), we measure the employment gap in two ways. *GAP1* is an indicator variable that equals one if an executive worked in other listed firms during the gap between his appointments in two different firms, and zero otherwise.^4^ *GAP2* is an indicator variable that equals one if the employment gap year is greater than or equal to the 75th percentile of the full sample, and zero otherwise. We apply DIB internal control index which is a comprehensive measure of the internal control quality of Chinese listed firms. The DIB internal control index is formulated by a third-party professional rating agency based on the internal control disclosure and assessment of Chinese listed firms (Li et al., 2020). As a composite index of COSO’s five specific elements, this index is widely used in Chinese studies to measure the efficiency and effectiveness of internal control adoption (Wang et al., 2018; Chen et al., 2019). To compare the internal control level of the predecessor and the successor firm, we define *ICQ* as the difference between the internal control index (taking the natural logarithm) of the predecessor and the successor firm. A higher *ICQ* means that the successor firm’s internal control system is more effective than the predecessor firm.

#### Control Variables

We control for a number of firm-level and individual-level variables in the regression models according to prior literature on CSR performance. For firm-level controls, we include *SIZE* (=natural logarithm of total assets) as larger firms face greater public pressure to take social responsibility (Dhaliwal et al., 2011). We include *LEV* (=the ratio of total debts to total assets) since a firm’s debts usually play a monitoring role (Leftwich et al., 1981). As firms with higher profitability and better financial performance have more resources to practice CSR activities (McGuire et al., 1988; Pava and Krausz, 1996), we include *ROA* (=the ratio of net profit to total assets) and *AGE* [=natural logarithm of (one plus) the number of years since the firm was established]. As state-owned firms have an obligation to participate in CSR activities (Chang et al., 2021), we include *SOE* (an indicator variable that equals one if the firm is controlled by the central or local government, and zero otherwise). Following Hussain et al. (2018), we control for corporate governance variables such as *BOARD* (=natural logarithm of the number of directors on board) and *INDDIR* (=the ratio of the number of independent directors to the number of directors on board).

For individual-level controls, in line with prior literature (Manner, 2010; Tang et al., 2015; Yuan et al., 2019), we include executives’ personal characteristics such as *GENDER* (an indicator variable that equals one if the executive is male, and zero otherwise), *OLD* [=natural logarithm of (one plus) the age of the executive], and *EDUC* (an indicator variable that equals one if the executive has a postgraduate degree, and zero otherwise). We also include *POSITION* (an indicator variable that equals one if the executive is the firm’s chairman, CEO and CFO, and zero otherwise) to examine whether core executives have a stronger impact on CSR performance.

To minimize the effect of a firm’s CSR-related decisions in the recent past (Chin et al., 2013; Tang et al., 2015), we also control for the CSR performance of the successor firm before the appointment of the new executive (*PRECSR_S/PRECSR_R*). Although we have controlled many variables that may affect CSR performance according to existing literature, there may still be endogeneity problems due to missing variables. Therefore, we use firm fixed effects (*FE*_*FIRM*_) to control for unobserved, time-invariant corporate characteristics. We also include year fixed effects (*FE*_*YEAR*_) to control for time-varying factors that affect CSR performance^5^. For some models that are not applicable to control for firm fixed effects, we include industry fixed effects (*FE*_*IND*_) and province fixed effects (*FE*_*PROV*_) to control for heterogeneity across industries and provinces. Table 2 shows the detailed variable description.

**TABLE 2:** Variable description.

Variable	Definition
*P_CSR_S*	The CSR score of the successor firm in the first full year of the executive’s employment (/100).
*P_CSR_R*	The CSR rating of the successor firm in the first full year of the executive’s employment. The CSR rating is ranging from A (highest) to E (lowest). We define the rating of A as 5, B as 4, and so on.
*F_CSR_S*	The CSR score of the predecessor firm in the last year of the executive’s employment (/100).
*F_CSR_R*	The CSR rating of the predecessor firm in the last year of the executive’s employment.
*GAP1*	Indicator variable that equals one if an executive worked in other listed firms during the gap between his appointments in two different firms, and zero otherwise.
*GAP2*	Indicator variable that equals one if the employment gap year is greater than or equal to the 75th percentile of the full sample, and zero otherwise.
*ICQ*	Natural logarithm of the internal control index of the successor firm – Natural logarithm of the internal control index of the predecessor firm.
*SIZE*	Natural logarithm of total assets.
*LEV*	The ratio of total debts to total assets.
*ROA*	The ratio of net profit to total assets.
*AGE*	Natural logarithm of (one plus) the number of years since the firm was established.
*SOE*	Indicator variable that equals one if the firm is controlled by the central or local government, and zero otherwise.
*BOARD*	Natural logarithm of the number of directors on board.
*INDDIR*	The ratio of the number of independent directors to the number of directors on board.
*GENDER*	Indicator variable that equals one if the executive is male, and zero otherwise.
*OLD*	Natural logarithm of (one plus) the age of the executive.
*EDUC*	Indicator variable that equals one if the executive has a postgraduate degree, and zero otherwise.
*POSITION*	Indicator variable that equals one if the executive is the firm’s chairman, CEO and CFO, and zero otherwise.
*PRECSR_S*	The CSR score of the successor firm in the year before the new executive’s employment (/100).
*PRECSR_R*	The CSR rating of the successor firm in the year before the new executive’s employment.
*FE*_*FIRM*_	Firm fixed effects.
*FE*_*YEAR*_	Year fixed effects.
*FE*_*IND*_	Industry fixed effects.
*FE*_*PROV*_	Province fixed effects.

### Model Setting

To examine the relationship between the CSR performance of the predecessor and the successor firm in the context of inter-firm executive mobility, we construct the following model:


(1) P_CSR=α+0αF1_CSR+Controls+FEFIRM +FE+YEARε

The dependent variable *P_CSR* captures the CSR performance of the successor firm and the independent variable *F_CSR* captures the CSR performance of the predecessor firm. In this manuscript, the CSR score (*P_CSR_S/F_CSR_S*) and CSR rating (*P_CSR_R/F_CSR_R*) are selected as proxies of CSR performance. *Controls* are a vector of corporate and individual attributes that could affect a firm’s CSR performance. *FE*_*FIRM*_ and *FE*_*YEAR*_ capture fixed effects of firm and year, respectively. The Hypothesis 1 holds if the coefficient of α*_1_* is significantly positive.

To examine the moderating effect of the employment gap on the relationship between the CSR performance of the predecessor and the successor firm, we construct the following model:


(2) P_CSR=β+0βF1_CSR+βF2_CSR×GAP+βGAP3+Controls+FE+FIRMFE+YEARε

The moderator variable *GAP* has been discussed in section “Moderator Variables”. *F_CSR* × *GAP* is the interaction term of the CSR performance of the predecessor firm and the executive’s employment gap. Other variables are consistent with Model (1). The Hypothesis 2 holds if the coefficient of β*_2_* is significantly negative.

To examine the moderating effect of the internal control quality on the relationship between the CSR performance of the predecessor and the successor firm, we construct the following model:


(3) P_CSR=γ+0γF1_CSR+γF2_CSR×ICQ+γICQ3+Controls+FE+FIRMFE+YEARε

The moderator variable *ICQ* has been discussed in section “Moderator Variables”. *F_CSR* × *ICQ* is the interaction term of the CSR performance of the predecessor firm and the differences in the internal control quality between the predecessor and the successor firm. Other variables are consistent with Model (1). The Hypothesis 3 holds if the coefficient of γ*_2_* is significantly positive.

Since the interaction term in the moderating effect model is likely to covary with the separate terms to some extent, prior literature recommended the Mean-centering approach to alleviate collinearity related concerns (Cronbach, 1987). Therefore, we mean-center all independent variables that constitute the interaction term (*F_CSR*, *GAP*, and *ICQ*) in all moderating effect models in this manuscript to mitigate the potential threat of multicollinearity.

## Empirical Analysis and Results

### Summary Statistics

Table 3 reports the summary statistics of the main variables. To minimize the effect of outliers and ensure the right skewness, all continuous variables are winsorized at 1 and 99% levels. In our sample, the mean (median) of the successor firms’ CSR performance are 0.244 (0.231) for CSR score (*P_CSR_S*) and 2.099 (2.000) for CSR rating (*P_CSR_R*); the mean (median) of the predecessor firms’ CSR performance are 0.269 (0.224) for CSR score (*F_CSR_S*) and 2.253 (2.000) for CSR rating (*F_CSR_R*). It indicates that there is a great variation in the CSR performance of Chinese listed firms. For the moderator variables, 58.7% of sample executives had worked in other listed firms during the gap between his appointments in two different firms (*GAP1*) and 37.1% of sample executives’ employment gap is more than 3 years (75th percentile of the full sample) (*GAP2*). Moreover, the internal control quality of the successor firm is generally weaker than that of the predecessor firm. The descriptive statistics of control variables are similar to the previous literature.

**TABLE 3:** Summary statistics.

Variable	*N*	Mean	SD	Min	P25	P50	P75	Max
*P_CSR_S*	15,943	0.244	0.147	–0.045	0.176	0.231	0.281	0.746
*P_CSR_R*	15,943	2.099	0.542	1.000	2.000	2.000	2.000	5.000
*F_CSR_S*	15,943	0.269	0.192	–0.053	0.160	0.224	0.301	0.779
*F_CSR_R*	15,943	2.253	0.722	1.000	2.000	2.000	2.000	5.000
*GAP1*	15,943	0.587	0.492	0.000	0.000	1.000	1.000	1.000
*GAP2*	15,943	0.371	0.483	0.000	0.000	0.000	1.000	1.000
*ICQ*	10,567	–0.015	0.192	–0.745	–0.101	–0.012	0.078	0.677
*SIZE*	15,943	22.520	1.824	19.610	21.240	22.180	23.370	29.190
*LEV*	15,943	0.455	0.229	0.056	0.271	0.438	0.625	0.950
*ROA*	15,943	0.038	0.073	–0.367	0.014	0.039	0.072	0.203
*AGE*	15,943	2.871	0.341	1.946	2.639	2.944	3.135	3.526
*SOE*	15,943	0.135	0.342	0.000	0.000	0.000	0.000	1.000
*BOARD*	15,943	2.171	0.217	1.386	2.079	2.197	2.197	2.996
*INDDIR*	15,943	0.374	0.053	0.300	0.333	0.333	0.429	0.571
*GENDER*	15,943	0.854	0.354	0.000	1.000	1.000	1.000	1.000
*OLD*	15,943	3.966	0.145	3.584	3.871	3.970	4.060	4.290
*EDUC*	15,943	0.567	0.495	0.000	0.000	1.000	1.000	1.000
*POSITION*	15,943	0.114	0.318	0.000	0.000	0.000	0.000	1.000
*PRECSR_S*	15,943	0.246	0.161	–0.047	0.173	0.219	0.274	0.762
*PRECSR_R*	15,943	2.140	0.589	1.000	2.000	2.000	2.000	4.000

*The decrease in the number of observations of ICQ is due to the late start year of the internal control index provided in the database and the existence of some missing values.*

### Correlation Matrix

Table 4 reports the Pearson correlation matrix of the main variables. The significantly positive correlation between *P_CSR* and *F_CSR* indicates that the CSR performance of the predecessor firm is positively associated with that of the successor firm, supporting H1 preliminarily. We also find that the *PRECSR* is significantly positively correlated with *P_CSR* which documents that the CSR performance exhibits strong inertia. To further test the existence of multicollinearity, we calculate the variance inflation factor (VIF) for regression variables. All the VIFs are less than 4 which are well below the acceptable limit (Kennedy, 1998), indicating that there is no serious multicollinearity problem in our study.

**TABLE 4:** Pearson correlation matrix.

	(1)	(2)	(3)	(4)	(5)	(6)	(7)	(8)	(9)	(10)	(11)	(12)	(13)	(14)	(15)	(16)
(1)*P_CSR_R*	1															
(2)*F_CSR_R*	0.133^a^	1														
(3)*GAP1*	−0.065^a^	−0.019^b^	1													
(4)*GAP2*	−0.078^a^	0.123^a^	0.184^a^	1												
(5)*ICQ*	0.102^a^	−0.159^a^	–0.006	−0.082^a^	1											
(6)*SIZE*	0.242^a^	0.124^a^	0.010	0.015^c^	0.055^a^	1										
(7)*LEV*	0.067^a^	0.076^a^	−0.025^a^	–0.007	−0.054^a^	0.602^a^	1									
(8)*ROA*	0.223^a^	0.018^b^	0.024^a^	–0.007	0.251^a^	−0.091^a^	−0.400^a^	1								
(9)*AGE*	–0.010	–0.007	0.043^a^	0.056^a^	−0.022^b^	0.175^a^	0.194^a^	−0.123^a^	1							
(10)*SOE*	0.101^a^	0.047^a^	−0.051^a^	–0.009	0.021^b^	0.197^a^	0.114^a^	–0.004	0.048^a^	1						
(11)*BOARD*	0.162^a^	0.077^a^	−0.039^a^	–0.012	0.008	0.456^a^	0.276^a^	−0.028^a^	0.083^a^	0.135^a^	1					
(12)*INDDIR*	–0.005	0.012	0.012	–0.005	−0.022^b^	−0.013^c^	–0.004	−0.019^b^	−0.013^c^	−0.044^a^	−0.493^a^	1				
(13)*GENDER*	0.010	0.026^a^	–0.005	0.009	–0.001	0.065^a^	0.041^a^	–0.006	−0.018^b^	0.019^b^	0.011	0.016^b^	1			
(14)*OLD*	−0.023^a^	0.042^a^	0.228^a^	0.109^a^	−0.025^b^	0.060^a^	0.001	0.025^a^	0.015^c^	0.021^a^	0.034^a^	–0.011	0.034^a^	1		
(15)*EDUC*	−0.016^b^	0.012	0.094^a^	0.034^a^	0.001	0.030^a^	−0.057^a^	0.029^a^	−0.100^a^	−0.025^a^	0.011	0.004	0.018^b^	−0.045^a^	1	
(16)*POSITION*	0.016^c^	0.022^a^	−0.249^a^	−0.037^a^	−0.024^b^	0.037^a^	0.053^a^	−0.068^a^	0.016^b^	0.049^a^	–0.007	0.015^c^	0.054^a^	−0.238^a^	−0.068^a^	1
(17)*PRECSR_R*	0.406^a^	0.117^a^	−0.041^a^	−0.056^a^	0.041^a^	0.289^a^	0.108^a^	0.078^a^	0.030^a^	0.063^a^	0.184^a^	−0.020^b^	0.021^a^	–0.010	−0.036^a^	−0.023^a^

*^a,b, and c^ represent significance at the 1, 5, and 10% levels, respectively (two-tailed). The CSR score variables are highly positively correlated with the CSR rating variables (Pearson correlation = 0.869, p < 0.01). For the sake of brevity, we only present the CSR rating variables in the correlation matrix.*

### Regression Results

#### Test of Hypothesis 1

Table 5 reports the regression results of Model (1). The dependent variables in Columns (1) and (2) are *P_CSR_S* and *P_CSR_R*, respectively. There is a positive and significant association between the CSR performance of the predecessor firm in the last year of an executive’s employment (*F_CSR_S/F_CSR_R*) and that of the successor firm in the first full year of an executive’s employment (*P_CSR_S/P_CSR_R*) after controlling other variables, especially previous CSR performance of the successor firm. It indicates that when an executive moves to another firm, his value and preference for CSR reflected in the predecessor firm will maintain consistency and lead to similar decisions and practices about CSR at the successor firm, which is consistent with H1.

**TABLE 5:** Regression results of Model (1).

Dep. Var:	(1)	(2)	(3)	(4)
	
	Linear model		Tobit model	Order logit model
	
	*P_CSR_S*	*P_CSR_R*	*P_CSR_S*	*P_CSR_R*
	
	Coef. (*t*-stat.)	Coef. (*t*-stat.)	Coef. (*t*-stat.)	Coef. (*z*-stat.)
*F_CSR_S*	0.010***		0.021***	
	(2.716)		(3.858)	
*F_CSR_R*		0.010**		0.072*
		(2.379)		(1.855)
*SIZE*	0.025***	0.045***	0.013***	0.327***
	(10.312)	(4.243)	(7.133)	(12.272)
*LEV*	−0.025**	–0.066	–0.017	–0.300
	(−2.500)	(−1.513)	(−1.525)	(−1.610)
*ROA*	0.531***	1.370***	0.837***	13.322***
	(32.681)	(19.023)	(21.690)	(26.040)
*AGE*	0.145***	0.912***	0.005	0.050
	(7.267)	(10.275)	(0.863)	(0.546)
*SOE*	0.026***	0.062***	0.019***	0.534***
	(6.663)	(3.570)	(2.940)	(6.229)
*BOARD*	0.003	0.176***	0.023*	0.546***
	(0.240)	(3.800)	(1.921)	(3.275)
*INDDIR*	0.188***	0.937***	0.047	0.333
	(5.866)	(6.592)	(1.132)	(0.536)
*GENDER*	–0.002	−0.021**	–0.004	−0.183**
	(−1.046)	(−2.080)	(−1.275)	(−2.202)
*OLD*	–0.006	–0.029	–0.009	−0.487**
	(−1.056)	(−1.162)	(−1.076)	(−2.390)
*EDUC*	−0.004**	−0.021**	0.003	−0.137**
	(−2.221)	(−2.462)	(1.096)	(−2.337)
*POSITION*	0.007***	0.033***	0.005	0.053
	(3.039)	(3.091)	(1.209)	(0.591)
*PRECSR_S*	0.181***		0.351***	
	(23.685)		(20.354)	
*PRECSR_R*		0.160***		1.371***
		(19.365)		(30.088)
*FE*_*FIRM*_	*Yes*	*Yes*	*No*	*No*
*FE*_*YEAR*_	*Yes*	*Yes*	*Yes*	*Yes*
*FE*_*IND*_	*No*	*No*	*Yes*	*Yes*
*FE*_*PROV*_	*No*	*No*	*Yes*	*Yes*
*N*	15,943	15,943	16,480	16,480
*R^2^/Pseudo R^2^*	0.775	0.675	–0.913	0.345

***, **, and * represent significance at the 1, 5, and 10% levels, respectively (two-tailed). The increase in the number of observations in Columns (3) and (4) is due to the fact that these models do not control for firm fixed effects and thus do not exclude singleton observations.

Consistent with previous literature (Manner, 2010; Dhaliwal et al., 2011; Tang et al., 2015; Hussain et al., 2018; Chang et al., 2021), we find that firms with larger scales (*SIZE*), stronger profitability (*ROA*), longer operating years (*AGE*), state-owned property (*SOE*), more independent directors (*INDDIR*), and better CSR performance in the past (*PRECSR_S/PRECSR_R*) have better CSR performance. For individual-level controls, we find that male (*GENDER*) and highly educated (*EDUC*) executives are less likely to engage in proactive CSR practices, and senior executives (*POSITION*) have a stronger impact on the CSR performance.

As the dependent variable, *P_CSR_S*, is bounded between zero and one, and the other dependent variable, *P_CSR_R*, is an ordinal number from one to five, we also estimate a Tobit regression and an Order Logit regression for Model (1) to check the robustness, respectively.^6^ The results in Columns (3) and (4) show that our findings are robust to these alternative estimation techniques.

#### Test of Hypothesis 2

Table 6 reports the regression results of Model (2). Columns (1) and (3) present the moderating effect of the work experience during the employment gap (*GAP1*). The results show that the coefficients of *F_CSR_S* and *F_CSR_R* are still significantly positive, and the coefficients of interaction terms *F_CSR_S* × *GAP* and *F_CSR_R* × *GAP* are significantly negative. Similar results can be found in Columns (2) and (4) which present the moderating effect of the length of the employment gap (*GAP2*). The above results indicate that in the context of executives’ inter-firm mobility, a longer employment gap would inhibit the executives’ behavioral consistency on the CSR fulfillment, which in turn weakens the positive association between the CSR performance of the predecessor and the successor firm, which is consistent with H2.

**TABLE 6:** Regression results of Model (2).

Dep. Var:	(1)	(2)	(3)	(4)
	
	*P_CSR_S*		*P_CSR_R*	
	*GAP* = *GAP1*	*GAP* = *GAP2*	*GAP* = *GAP1*	*GAP* = *GAP2*
	
	Coef.	Coef.	Coef.	Coef.
	(*t*-stat.)	(*t*-stat.)	(*t*-stat.)	(*t*-stat.)
*F_CSR_S*	0.010***	0.012***		
	(2.828)	(3.159)		
*F_CSR_S* × *GAP*	−0.024***	−0.020***		
	(−3.305)	(−2.738)		
*F_CSR_R*			0.011**	0.015***
			(2.519)	(3.427)
*F_CSR_R* × *GAP*			−0.034***	−0.041***
			(−4.053)	(−4.853)
*GAP*	0.001	–0.001	–0.008	–0.008
	(0.457)	(−0.402)	(−1.142)	(−1.177)
*SIZE*	0.025***	0.025***	0.044***	0.043***
	(10.288)	(10.257)	(4.189)	(4.128)
*LEV*	−0.025**	−0.025**	–0.065	–0.064
	(−2.515)	(−2.488)	(−1.499)	(−1.478)
*ROA*	0.531***	0.532***	1.368***	1.373***
	(32.638)	(32.693)	(19.012)	(19.085)
*AGE*	0.145***	0.145***	0.908***	0.905***
	(7.238)	(7.263)	(10.244)	(10.205)
*SOE*	0.026***	0.026***	0.062***	0.061***
	(6.651)	(6.628)	(3.532)	(3.512)
*BOARD*	0.002	0.002	0.175***	0.172***
	(0.196)	(0.195)	(3.780)	(3.709)
*INDDIR*	0.188***	0.186***	0.940***	0.923***
	(5.864)	(5.814)	(6.622)	(6.505)
*GENDER*	–0.002	–0.002	−0.021**	−0.020**
	(−1.077)	(−1.020)	(−2.098)	(−2.045)
*OLD*	–0.007	–0.006	–0.029	–0.028
	(−1.255)	(−1.082)	(−1.152)	(−1.125)
*EDUC*	−0.004**	−0.004**	−0.020**	−0.021**
	(−2.265)	(−2.213)	(−2.386)	(−2.438)
*POSITION*	0.007***	0.007***	0.030***	0.032***
	(3.068)	(3.001)	(2.838)	(3.048)
*PRECSR_S*	0.180***	0.180***		
	(23.548)	(23.614)		
*PRECSR_R*			0.158***	0.159***
			(19.198)	(19.293)
*FE*_*FIRM*_	*Yes*	*Yes*	*Yes*	*Yes*
*FE*_*YEAR*_	*Yes*	*Yes*	*Yes*	*Yes*
*N*	15,943	15,943	15,943	15,943
*R* ^2^	0.775	0.775	0.675	0.676

*** and ** represent significance at the 1 and 5% levels, respectively (two-tailed). All independent variables that constitute the interaction term are mean-centered to mitigate the potential threat of multicollinearity.

#### Test of Hypothesis 3

Table 7 reports the regression results of Model (3). Columns (1) and (2) present the moderating effect of the differences in the internal control quality between the predecessor and the successor firm (*ICQ*). The results show that the coefficients of *F_CSR_S* and *F_CSR_R* are still significantly positive, and the coefficients of interaction terms *F_CSR_S* × *ICQ* and *F_CSR_R* × *ICQ* are significantly positive. The above results indicate that the successor firm’s more effective internal control system than the predecessor firm could reduce executives’ risk-taking behaviors and raise their initiatives to engage in CSR activities in the new working environment, which in turn strengthens the positive association between the CSR performance of the predecessor and the successor firm, which is consistent with H3.

**TABLE 7:** Regression results of Model (3).

Dep. Var:	(1)	(2)
	
	*P_CSR_S*	*P_CSR_R*
	
	Coef.	Coef.
	(*t*-stat.)	(*t*-stat.)
*F_CSR_S*	0.016***	
	(3.198)	
*F_CSR_S* × *ICQ*	0.062***	
	(2.582)	
*F_CSR_R*		0.016***
		(2.719)
*F_CSR_R* × *ICQ*		0.104***
		(3.622)
*ICQ*	0.010*	−0.003
	(1.672)	(−0.118)
*SIZE*	0.029***	0.029*
	(8.588)	(1.910)
*LEV*	−0.060***	−0.267***
	(−4.066)	(−4.097)
*ROA*	0.576***	1.322***
	(21.705)	(11.179)
*AGE*	0.160***	0.882***
	(6.138)	(7.560)
*SOE*	0.027***	0.070***
	(5.531)	(3.230)
*BOARD*	−0.016	0.075
	(−1.184)	(1.248)
*INDDIR*	0.085**	0.578***
	(2.071)	(3.165)
*GENDER*	−0.000	−0.011
	(−0.127)	(−0.829)
*OLD*	−0.002	−0.005
	(−0.256)	(−0.134)
*EDUC*	0.006*	0.024*
	(1.931)	(1.667)
*POSITION*	−0.008***	−0.035***
	(−3.098)	(−2.906)
*PRECSR_S*	0.187***	
	(19.531)	
*PRECSR_R*		0.182***
		(17.913)
*FE*_*FIRM*_	*Yes*	*Yes*
*FE*_*YEAR*_	*Yes*	*Yes*
*N*	10,567	10,567
*R* ^2^	0.779	0.683

***, **, and * represent significance at the 1, 5, and 10% levels, respectively (two-tailed). All independent variables that constitute the interaction term are mean-centered to mitigate the potential threat of multicollinearity. The decrease in the number of observations is due to the late start year of the internal control index provided in the database and the existence of some missing values.

## Additional Analyses and Robustness Tests

### Subsample Tests

In this section, we conduct three subsample tests. First, due to the particularity of ST, delisted and financial industry firms, we exclude these firms’ observations and re-examine Model (1) to ensure the quality of the data (Jiang et al., 2022). The results in Columns (1–3) of Table 8 show that our findings are robust.

**TABLE 8:** Subsample tests.

Dep. Var:	(1)	(2)	(3)	(4)	(5)	(6)	(7)	(8)	(9)
	
	Exclude ST, delisted, and financial industry firms			Exclude executives re-employed by the same firm			Sub-sample of core executives		
	Coef.	Coef.	Coef.	Coef.	Coef.	Coef.	Coef.	Coef.	Coef.
*P_CSR*	(*t*-stat.)	(*t*-stat.)	(*t*-stat.)	(*t*-stat.)	(*t*-stat.)	(*t*-stat.)	(*t*-stat.)	(*t*-stat.)	(*t*-stat.)
*F_CSR*	0.009**	0.010**	0.016***	0.013***	0.014***	0.021***	0.010**	0.012***	0.016***
	(2.084)	(2.282)	(2.617)	(3.064)	(3.218)	(3.386)	(2.358)	(2.737)	(2.649)
*F_CSR* × *GAP*		−0.035***			−0.031***			−0.031***	
		(−4.043)			(−3.622)			(−3.476)	
*F_CSR* × *ICQ*			0.103***			0.101***			0.087***
			(3.465)			(3.417)			(2.953)
*Controls*	*Yes*	*Yes*	*Yes*	*Yes*	*Yes*	*Yes*	*Yes*	*Yes*	*Yes*
*FE*_*FIRM*_	*Yes*	*Yes*	*Yes*	*Yes*	*Yes*	*Yes*	*Yes*	*Yes*	*Yes*
*FE*_*YEAR*_	*Yes*	*Yes*	*Yes*	*Yes*	*Yes*	*Yes*	*Yes*	*Yes*	*Yes*
*N*	15,010	15,010	10,006	15,311	15,311	10,000	14,360	14,360	9,489
*R* ^2^	0.674	0.675	0.684	0.678	0.679	0.687	0.691	0.692	0.704

*** and ** represent significance at the 1 and 5% levels, respectively (two-tailed). All independent variables that constitute the interaction term are mean-centered to mitigate the potential threat of multicollinearity. For the sake of brevity, we only present the results of CSR rating variables (P_CSR = P_CSR_R, F_CSR = F_CSR_R) and the first proxy of employment gap (GAP = GAP1).

Second, when executives are re-employed by the firms that they once worked in, the association of CSR performance between the predecessor and the successor firm may be caused by the highly similar background of the same firm at different times. Therefore, we exclude observations that the predecessor and the successor firm are the same one to ensure that the executive was appointed by a different firm. The results in Columns (4–6) of Table 8 show that our findings are robust.

Third, compared with general executives, the core executives may pose a greater influence on CSR fulfillment decisions. Therefore, we limit our sample to the executives who serve as the firm’s chairman, CEO, CFO, and board of directors in the successor firm. The results in Columns (7–9) of Table 8 show that our findings are robust.^7^

### Heckman Two-Stage Model

Since the research design of this manuscript restricts the sample to executives who worked in at least two listed firms, it only contains executives who left a firm and then were employed by another firm. In fact, there are still a lot of executives who never changed their positions or were not employed by another listed firm after leaving the previous position, which might make our sample have self-selection problems. Therefore, we use the Heckman two-stage model to address the possible problem of sample selection bias.

In the first stage, we construct a Probit model in which the dependent variable *REPOST* is an indicator variable that equals one if the executive was hired by another listed firm after he or she left the previous position, and zero otherwise. The independent variables include individual characteristics of executives such as *GENDER, OLD, EDUC, POSITION*, and characteristics of the predecessor firms such as *SIZE*, *ROA*, *LEV*, and *PRECSR*. As the development of the industry’s human capital market is also an important factor in executive recruitment, we include *PRMAR* (=change of the number of executives in an industry in year *t* divided by the number of executives in an industry in year *t*−1). We also control for the year, industry and province fixed effects. The unreported results show that executives are more likely to be hired by other listed firms if they are older males with higher educational level and their predecessor firm performed well in CSR.

In the second stage, we re-estimate Model (1–3) after adding the Inverse Mills Ratio (*IMR*) calculated in the first stage as a control variable. The results in Columns (1–3) of Table 9 show that our findings are robust when controlling the selection bias, which means that the previous findings of this manuscript are not influenced by selection bias.

**TABLE 9:** Heckman two-stage model (the second stage).

Dep. Var*: P_CSR*	(1)	(2)	(3)
	
	Coef.	Coef.	Coef.
	(*t*-stat.)	(*t*-stat.)	(*t*-stat.)
*F_CSR*	0.011**	0.012***	0.017***
	(2.542)	(2.698)	(2.891)
*F_CSR* × *GAP*		−0.036***	
		(−4.247)	
*F_CSR* × *ICQ*			0.107***
			(3.686)
*IMR*	0.025*	0.025*	0.037**
	(1.877)	(1.879)	(2.071)
*Controls*	*Yes*	*Yes*	*Yes*
*FE*_*FIRM*_	*Yes*	*Yes*	*Yes*
*FE*_*YEAR*_	*Yes*	*Yes*	*Yes*
*N*	15,552	15,552	10,330
*R* ^2^	0.675	0.675	0.683

***, **, and * represent significance at the 1, 5, and 10% levels, respectively (two-tailed). All independent variables that constitute the interaction term are mean-centered to mitigate the potential threat of multicollinearity. For the sake of brevity, we only present the results of CSR rating variables (P_CSR = P_CSR_R, F_CSR = F_CSR_R) and the first proxy of employment gap (GAP = GAP1). The decrease in the number of observations is due to the fact that some observations failed to obtain an IMR in the first stage regression.

### Propensity Score Matching Method

One alternative explanation of our findings is that the positive association of CSR performance between the predecessor and the successor firm is the result of similar corporate characteristics instead of the inter-firm executive mobility. Therefore, we adopt a PSM method to find a matching firm for each predecessor and successor firm according to *SIZE*, *LEV*, *ROA*, *AGE*, *BOARD*, and *INDDIR* in the same industry in the same year but without executive mobility. Then we repeat the regression for the successor firms and the predecessor firms’ matching sample, and for the predecessor firms and the successor firms’ matching sample, respectively. We expect that there is no longer a significantly positive association of CSR performance between these two groups of firms as they do not have the relevance caused by the change of the same executive.^8^

Table 10 reports the regression results of matching samples. In Columns (1–4), all coefficients of *F_CSR_S* and *F_CSR_R* are no longer significant, indicating that when two groups of firms no longer have the same executive as a connection, the CSR performance of the predecessor firm’s matching sample is not associated with the successor firm, as well as the CSR performance of the predecessor firm are not associated with the successor firm’s matching sample. This evidence further confirms the direct impact of individual executives’ value and preference for CSR on the CSR performance of the firm they served at.

**TABLE 10:** PSM method.

Dep. Var:	(1)	(2)	(3)	(4)
	
	Successor firms’ matching sample and predecessor firms		Successor firms and predecessor firms’ matching sample	
	*P_CSR_S*	*P_CSR_R*	*P_CSR_S*	*P_CSR_R*
	
	Coef.	Coef.	Coef.	Coef.
	(*t*-stat.)	(*t*-stat.)	(*t*-stat.)	(*t*-stat.)
*F_CSR_S*	0.001		0.001	
	(0.169)		(0.375)	
*F_CSR_R*		0.002		0.006
		(0.516)		(1.252)
*Controls*	*Yes*	*Yes*	*Yes*	*Yes*
*FE*_*FIRM*_	*Yes*	*Yes*	*Yes*	*Yes*
*FE*_*YEAR*_	*Yes*	*Yes*	*Yes*	*Yes*
*N*	15,079	15,079	14,580	14,580
*R* ^2^	0.808	0.726	0.778	0.678

*The decrease in the number of observations is due to the fact that some firms failed to find a matching firm under the PSM method.*

### Two-Stage Least Squares Approach

Although we have controlled for a series of crucial factors that affect firms’ CSR performance regarding prior studies, there may still be some omitted variables that may lead to biased model estimation results. To address the possible endogeneity issues, we re-estimate the regressions with the 2SLS approach. Following Bouslah et al. (2018) and Jia and Li (2022), we use the yearly average environmental performance score of listed firms in the same industry of the predecessor firms (*F_CSR_IND*) and the yearly average environmental performance score of listed firms in the same city of the predecessor firms (*F_CSR_CITY*) as the instrumental variables.

As environmental performance is an important part of CSR performance, it is expected that a firm’s CSR performance is closely related to the environmental performance of other companies in the same industry and in the same city. The results in Columns (1) and (3) of Table 11 show that the coefficients of both instrumental variables are positive and significant, indicating that it meets the relevance requirement for instrumental variables. Because the instrumental variables are measured by the environmental performance of listed firms in the same industry and city as the predecessor firms, and we limit the research sample of this part to the cross-industry as well as cross-city executive turnovers, *F_CSR_IND* and *F_CSR_CITY* are less likely to affect CSR performance of the successor firms in a completely different industry and city, plausibly satisfying the exclusion requirement.

**TABLE 11:** 2SLS approach.

Dep. Var:	(1)	(2)	(3)	(4)
	
	First stage	Second stage	First stage	Second stage
	
	*F_CSR_S*	*P_CSR_S*	*F_CSR_R*	*P_CSR_R*
	
	Coef.	Coef.	Coef.	Coef.
	(*t*-stat.)	(*t*-stat.)	(*t*-stat.)	(*t*-stat.)
*F_CSR_S (instrumented)*		0.055**		
		(2.42)		
*F_CSR_R (instrumented)*				0.039*
				(1.71)
*F_CSR_IND*	1.465***		7.357***	
	(8.71)		(11.60)	
*F_CSR_CITY*	1.064***		4.215***	
	(9.46)		(9.73)	
*Controls*	*Yes*	*Yes*	*Yes*	*Yes*
*FE*_*FIRM*_	*Yes*	*Yes*	*Yes*	*Yes*
*FE*_*YEAR*_	*Yes*	*Yes*	*Yes*	*Yes*
*N*	9,535	9,535	9,535	9,535
*R* ^2^	0.324	0.806	0.330	0.723

***, **, and * represent significance at the 1, 5, and 10% levels, respectively (two-tailed). The decrease in the number of observations is due to the fact that we limit the research sample of this part to the cross-industry as well as cross-city executive turnovers.

To further ensure the strength of our instrumental variables, we conduct an over-identification test, the Hansen J statistic results in a *p*-value of 0.760 and 0.929, suggesting that our instrumental variables do not exhibit over-identification. The under-identification test shows a *p*-value of zero, which means that there is no under-identification problem in the estimated results. The Cragg-Donald Wald *F* statistic is significant at the 1% level, thus rejecting the null hypothesis of weak instrumental variable. The above results indicate that the instrumental variables selected in this manuscript are reasonable and valid.

Columns (2) and (4) of Table 11 report the second-stage regression results. We find that our findings are robust when controlling the potential endogeneity problems using the 2SLS approach.

## Conclusion

Corporate social responsibility has important strategic implications for listed firms. Well-performed socially responsible activities can help listed firms create a good reputation and public image, establish valuable stakeholder relationships, and promote the long-term development of the firm. As the decision-maker of listed firms, executives’ value and preference have an essential impact on CSR fulfillment. Based on behavioral consistency theory, we examine the association of CSR performance among multiple firms for the same executive served at different times. By tracking the movement of executives across Chinese listed firms over the period 2010–2019, we find that there is a significantly positive association between the predecessor and the successor firm’s CSR performance for the same executive, implying that an individual’s value and preference for CSR maintain consistency within a certain period of time, and the association is influenced by executives’ employment gap and corporate internal control quality.

Our study provides a new perspective for the corporate governance research based on inter-firm executive mobility and highlights the impact of executives’ past experience in CSR practice on the CSR performance of other firms, enriching the literature on CSR determinants from the perspective of individual executives and behavioral consistency theory. In practice, our findings also have significant implications for the design of appropriate employment and talent evaluation system for listed firms. On the one hand, listed firms should improve the recruitment mechanisms in hiring executives, especially pay attention to their previous work experience and performance in CSR. On the other hand, a complete system for evaluating executives’ performance should be established which not only focuses on the firm’s accounting and stock performance, but also takes the CSR performance into consideration. In addition, a good corporate governance system can also provide an effective guarantee for the implementation of executives’ CSR decisions.

Future research could focus on the impact of executives’ other aspects of work experience and performance on their competitiveness in the labor market. Further, it would be important and interesting to investigate how listed firms make trade-offs when facing executive candidates with different competitive advantages, and how they choose executives that are more suitable for themselves.

## Data Availability Statement

Data used in this study are available from public sources identified in the study, further inquiries can be directed to the corresponding author.

## Author Contributions

The manuscript was written with the contributions of both authors. JW put forth great effort with regard to the project administration and the funding acquisition. JC performed the data curation and wrote the first draft. Both authors approved the final manuscript.

## Conflict of Interest

The authors declare that the research was conducted in the absence of any commercial or financial relationships that could be construed as a potential conflict of interest.

## Publisher’s Note

All claims expressed in this article are solely those of the authors and do not necessarily represent those of their affiliated organizations, or those of the publisher, the editors and the reviewers. Any product that may be evaluated in this article, or claim that may be made by its manufacturer, is not guaranteed or endorsed by the publisher.

## Footnotes

1 Executives in this study refer to all members of the management team disclosed in the annual reports of listed firms.

2 We also exclude observations that the predecessor and the successor firm are the same one to check the robustness in section “Subsample Tests.”

3 To ensure the integrity of observations, when executives move between more than three firms, we match all these firms in pairs and identify sequential turnovers. For example, for an executive worked in firm A from 2000 to 2012, in firm B from 2013 to 2015, and in firm C from 2014 to the present, both “A to B” and “A to C” constitute our research sample.

4 Due to data limitations, we are unable to determine whether executives worked in other non-listed firms during the employment gap, but this would not have a systemic impact on the results of this manuscript.

5 It should be noted that the data of this study are not panel data in the strict sense because a firm may have multiple incoming executives in a given year. In spite of this, the data still have the characteristics of panel data as it contains different firms in different time periods. Therefore, we can eliminate the problem of omitted variable bias by setting dummy variables for all firms and all years separately and incorporating them into the regression model (Tao, 2007).

6 One shortcoming of the Tobit regression is that it cannot include firm fixed effects (Malmendier et al., 2011). Instead, we control for industry and province fixed effects in the Tobit Model, as well as in the Order Logit Model. Due to this limitation, we still use the Linear Model in the following analyses.

7 We also try to further limit the sample to the chairman and CEO who are the most central decision-makers of listed firms. However, the remaining sample size is too small (*N* = 570) which may affect the efficiency of empirical tests. In spite of this limitation, we still find our findings are robust in untabulated results.

8 We do not conduct this robustness test for moderating effect model because there is no executive turnover between the two firms under the PSM method.
